# Chemical and kinetic insights into the Thermal Decomposition of an Oxide Layer on Si(111) from Millisecond Photoelectron Spectroscopy

**DOI:** 10.1038/s41598-017-14532-4

**Published:** 2017-10-27

**Authors:** J.-J. Gallet, M. G. Silly, M. El Kazzi, F. Bournel, F. Sirotti, F. Rochet

**Affiliations:** 1 Sorbonne Universités, UPMC Univ. Paris 06, and CNRS UMR 7614, Laboratoire de Chimie Physique Matière et Rayonnement (LCPMR), F-75005 Paris, France; 2 Synchrotron SOLEIL, L’Orme des Merisiers, Saint-Aubin, BP 48, F-91192 Gif-sur-Yvette, France; 3 Laboratoire de Physique de la Matière Condensée, CNRS and Ecole Polytechnique, Université Paris Saclay, F- 91128 Palaiseau, France; 4 Present Address: Paul Scherrer Institut, 5232 Villigen-PSI, Switzerland

## Abstract

Despite thermal silicon oxide desorption is a basic operation in semiconductor nanotechnology, its detailed chemical analysis has not been yet realized via time-resolved photoemission. Using an advanced acquisition system and synchrotron radiation, heating schedules with velocities as high as 100 K.s^−1^ were implemented and highly resolved Si 2p spectra in the tens of millisecond range were obtained. Starting from a Si(111)-7 × 7 surface oxidized in O_2_ at room temperature (1.4 monolayer of oxygen), changes in the Si 2p spectral shape enabled a detailed chemical analysis of the oxygen redistribution at the surface and of the nucleation, growth and reconstruction of the clean silicon areas. As desorption is an inhomogeneous surface process, the Avrami formalism was adapted to oxide desorption via an original mathematical analysis. The extracted kinetic parameters (the Avrami exponent equal to ~2, the activation energy of ~4.1 eV and a characteristic frequency) were found remarkably stable within a wide (~110 K) desorption temperature window, showing that the Avrami analysis is robust. Both the chemical and kinetic information collected from this experiment can find useful applications when desorption of the oxide layer is a fundamental step in nanofabrication processes on silicon surfaces.

## Introduction

Since the earlier experimental works of D’Evelyn *et al*.^1^ and Engstrom *et al*.^2^ the study of the thermal decomposition of silicon oxide layers on silicon has remained an active field of research, both at experimental^3–17^ and theoretical^18–21^ levels. Indeed, volatile SiO (SiO_g_) production is a key feature of nanoelectronics processes, naturally relative to silicon surface cleaning^22,23^, but also to nano-fabrication^24–28^. Microscopy studies pointed to a spatially inhomogeneous process both on Si(001)^9–11,14,15,13^ and (111) surfaces^12,16^. Clean voids appear randomly on the oxidized surface, leaving oxide patches whose thickness does not change, while surface pitting is observed in the clean areas. The later observation suggests that silicon atoms from the clean areas are consumed in the reaction and that clean areas grow via reaction of silicon monomers reaching the void periphery where they react with the oxide to form SiO_g_.

Consequently, four steps for oxide decomposition are envisaged^9,10,13,16^:

- creation of a mobile Si monomer (step 1);
- diffusion to the void boundary (step 2);
- reaction of mobile Si with SiO_2_ at the void boundary to form SiO_*x*_ suboxide species precursor to desorption (step 3);
- desorption of SiO_g_ (step 4).

**Figure 1:**
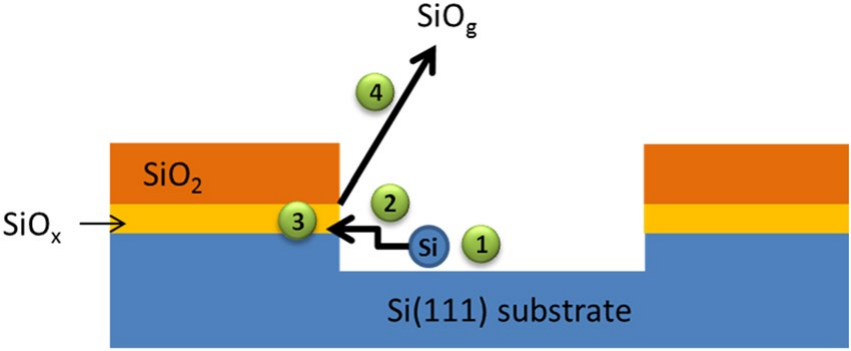
Schematic illustration of the thermal decomposition of oxide layers mediated by silicon monomer formation (step 1), surface mass transport (step 2) and formation of gaseous SiO (SiO_g_) (steps 3 and 4) by reaction at the periphery of the clean areas. SiO_x_ represents the interface suboxide layer (Si^3+^, Si^2+^ and Si^1+^) and SiO_2_ the fully oxidized silicon atoms (Si^4+^).

The thermal decomposition of ultra-thin oxidized layers (<1 ML of oxygen) may be limited by step 1^9,10^, while thicker thermal oxide layers are apparently limited by steps 3 or 4^13,16,29^. The void nucleation process itself may depend on the oxide thickness: the density can be constant with time (ultra-thin films^9^), or increase with time for nm-thick thermal films^13^. A pictorial description of the processes occurring during spatially inhomogeneous desorption is given in Fig. 1.

Most thermally programmed desorption (TPD) studies^1,2,7^ were based on the monitoring of the oxygen surface density via the measurement of the volatile SiO_g_ signal. With the notable exception of ref.^7^, they did not consider explicitly the spatially inhomogenous nature of the process. In particular, Engstrom *et al*.^2^ interpreted their TPD data in the framework of the Taylor-Weinberg method^30^, that uses a set of varying velocity heating schedules to extract kinetic data. The Taylor-Weinberg analysis is based on the usual Polanyi-Wigner expression of the reaction rate. At a given coverage, Arrhenius plots provide the value of the activation barrier and of a pseudo-zeroth order pre-factor that are both functions of coverage. One can legitimately wonder whether forcing data from a heterogeneous system into an order-based model (adapted to homogeneous kinetics) is appropriate^31^. Moreover, the analysis made by Engstrom *et al*.^2^ indicates there is significant compensation occurring^32,33^, which casts some doubts about the validity of the method. In contrast, Kinefuchi *et al*.^7^ took explicitly the inhomogeneous nature of the desorption process into account by considering the Avrami model, which is widely used to analyze the kinetics of phase transition^34–38^. The Avrami approach considers that the phase transformation (here the de-oxidation process) occurs by nucleation and growth of transformed areas (the clean Si).

TPD experiments that were carried out so far examined the evolution of the oxygen surface density, without going into the details of the chemical bonds appearing or disappearing during the desorption event. In contrast, core-level X-ray photoelectron spectroscopy (XPS) is in principle the ideal technique to study surface kinetics, as it allows an analysis both qualitative (via the binding energy shifts) and quantitative (via the photoemission intensity) of the species present on the surface at a given time, especially when the synchrotron radiation is used, as the high photon flux combined to a fast detection system^39^ enables the real-time monitoring of reactions with high time resolution. The present Temperature Programed XPS (TPXPS) study examines in real-time the decomposition of a controlled oxide film formed at room temperature on the Si(111)-7 × 7 surface after exposure to O_2_, corresponding to a coverage of 1.4 monolayer (ML). The thermal transformation is monitored via Si 2p photoemission in surface sensitive conditions (see Methods, section “Real-time Photoemission”), because it gives access to the chemical bonding at oxidized silicon interfaces^40–42^, via the identification of the four oxide states^40,42^. Moreover information on the clean areas comes from the appearance of the restatom (RA) component^43^. Therefore spectroscopic information on the clean areas can be provided independently of the SiO_g_ desorption event.

We adopt a non-isothermal^38^ approach, with average temperature velocities ${v}_{av}$ differing by an order of magnitude, between 2.5 K.s^−1^ and 100 K.s^−1^. Such high temperature velocities are permitted by a short acquisition time of 25 ms per spectrum and a high sampling frequency of 20 Hz (see section S1 in Supplementary Information). A photoemission experiment at this pace is uncommon^44^. It makes the present temperature velocities comparable to those of TPD experiments with quadrupole mass analyzers, for which temperature ramps up to hundreds of K.s^−1^ are not unusual. Such temperature schedules are possible because of the high signal/background ratio for Si 2p core level spectra, and the combination of the fast photoemission detection system synchronized to the temperature measurement, as well as to the electronically controlled annealing process. Similar experiments are currently performed with a time resolution of a few hundreds of millisecond^45^ using the same detection scheme as ours. Higher speed or better statistics should be achieved with the more complex parallel detection of ref.^46^, capable to handle higher count rates.

For any activated process, a large variation in the ramp velocities, translates to a large temperature window where the transformation (here, surface cleaning) is accomplished. This exploration in temperature enables the identification of chemical reactions that precede, accompany or are responsible for desorption, as their activation energies may differ. This also leads to a more accurate extraction of the kinetic parameters of a properly identified reaction.

After a qualitative chemical analysis of the transformations occurring at the surface (before and after oxide desorption), we perform a kinetic analysis of the desorption event itself based on the Avrami model. The parameter we follow is the fraction of non-transformed matter $\theta$, i.e. the oxide area fraction, using the Si^4+^ (SiO_2_) component intensity as a proxy. We give a mathematical treatment of the non-transformed fraction dependence on temperature, including the non-constancy of the temperature ramp velocity. In principle, the kinetic parameters can be obtained from one non-isothermal experiment, as in ref.^7^, but the use of various heating schedules helps testing the *robustness* of the Avrami analysis and the *stability* of the kinetic parameters in a wide range of desorption peak temperatures, that encompasses, in particular, the order-disorder 7 × 7 to “1 × 1” phase transition temperature of the clean surface^47,48^.

Finally, the increasing technological relevance of the Si(111) surface^49–51^ is a further motivation to gain a better chemical understanding and a robust parametrization of the spatially inhomogeneous de-oxidation process, as it can naturally create new opportunities of implementing maskless lithography^27,28^.

## Results

### Spectral analysis

**Figure 2:**
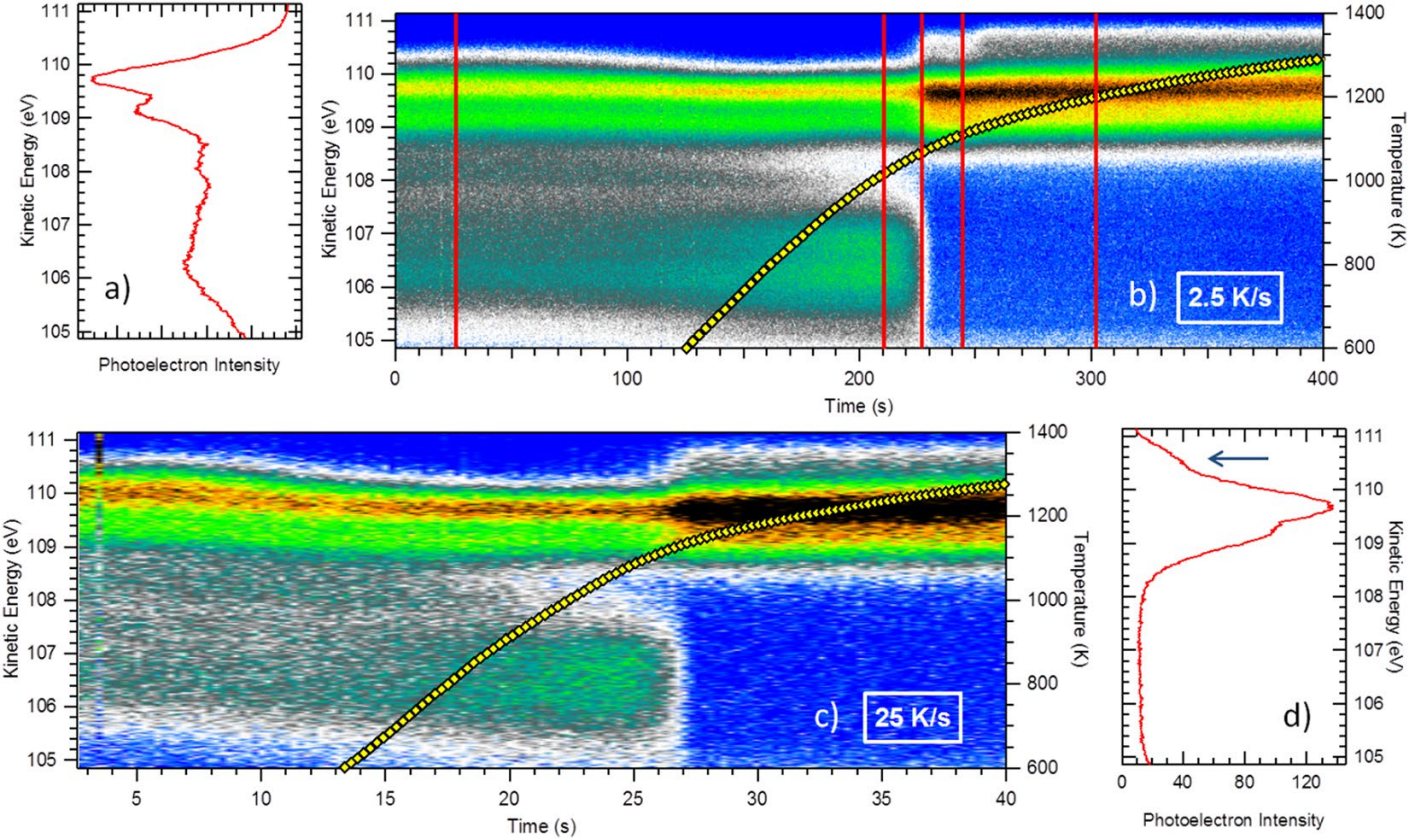
The Si 2p photoemission intensity (raw data, not corrected from the detector response) measured during the thermal annealing process (*v*_*av*_ = 2.5 K.s^−1^ in (**b**), and *v*_*av*_ = 25 K.s^−1^ in (**c**)) is represented in a color scale as function of time (horizontal axes) and of kinetic energy (vertical left axes). Panel (a) corresponds to the initial situation, i.e. the 7 × 7 surface oxidized at room temperature (1.4 O ML). The spectral weight below a kinetic energy of ~108.5 eV corresponds to the oxidation states. In (**d**) we show the de-oxidized surface at ~1135 K. The Si 2p core-level is actually a doublet with a spin-orbit splitting of 0.61 eV and a 2p_3/2_:2p_1/2_ branching ratio of 2. The blue arrow indicates the position of the restatom (RA) states of the cleaned surface. The temperature measured during the experiment with a calibrated pyrometer is indicated by the yellow line (right vertical scale). The vertical red solid lines in (**b**) corresponds to the selected spectra reported in Fig. 3. The photon energy is 210 eV, and the photoelectron emergence angle is 45° with respect to the surface normal. The temperature at time zero is 300 K. The lowest temperature measurable by the pyrometer is 600 K.

In Fig. 2, the 3D maps of the photoemission intensity (raw data, not corrected for detector response, see Methods) versus kinetic energy (left vertical axes) *and time* (bottom horizontal axes) are represented in a color scale for two temperature schedules with average heating velocities *v*_*av*_ = 2.5 K.s^−1^ (b) and *v*_*av*_ = 25 K.s^−1^ (c). The temperature evolution (right vertical axes) as a function of time is indicated by the yellow line in each case. The photoemission intensity versus kinetic energy spectra measured at the start (surface oxidized at room temperature) and at the end (de-oxidized surface, measured at 1135 K) of the heating process are presented in the panels (a) and (d), respectively. The latter two correspond to the integration of the 20 first and the 50 last spectra of the 3D maps, respectively.

**Figure 3:**
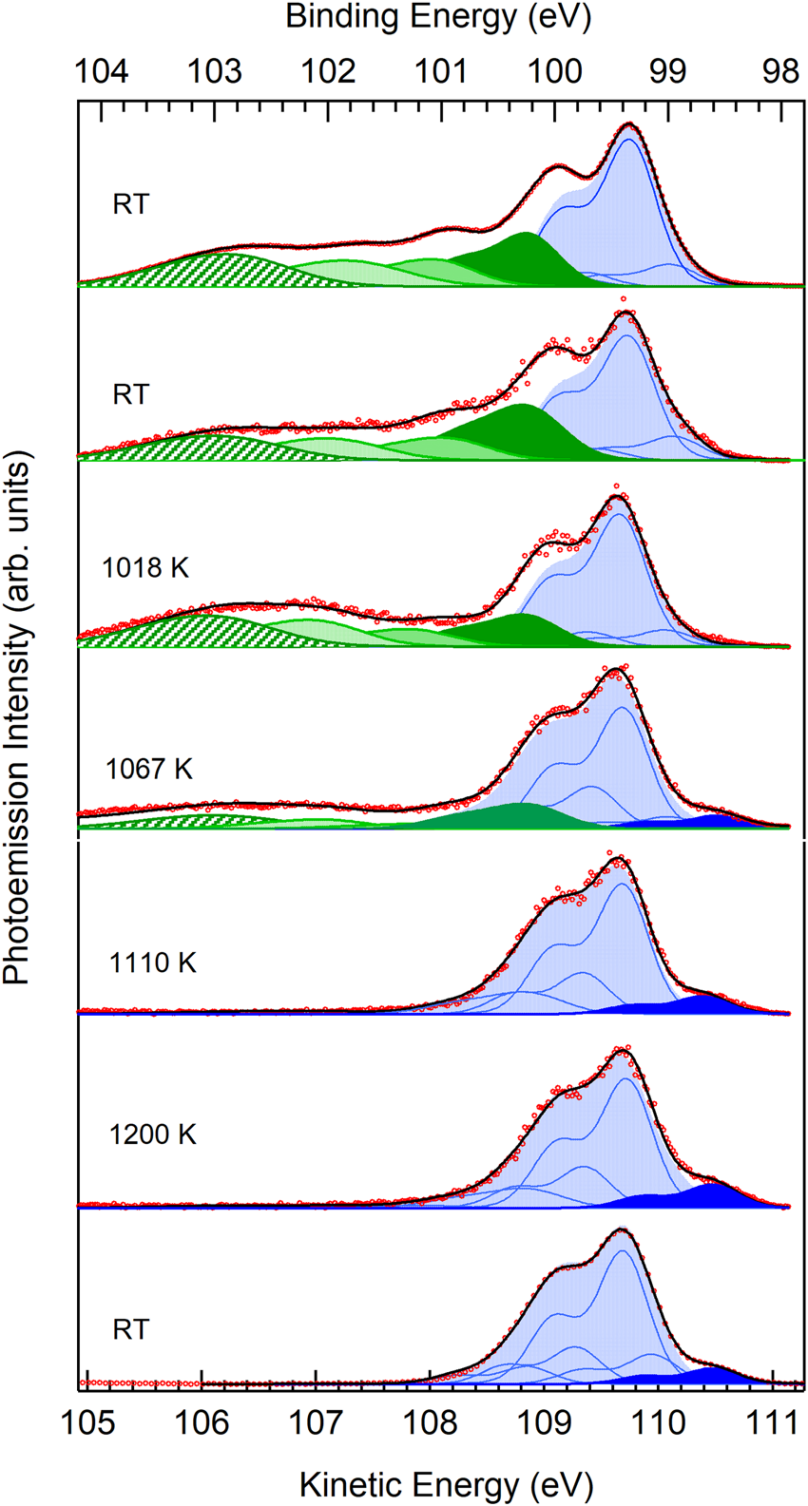
Selected spectra measured during the oxide layer desorption corresponding to the heating schedule (*v*_*av*_ = 2.5 K.s^−1^). The top and bottom spectra were acquired in sweep mode at room temperature. They correspond to the initial surface (1.4 ML of oxygen deposited at room temperature) exhibiting the contribution of the four oxidation states at higher binding energy (from dark green Si^1+^ to hatched green Si^4+^) than that of the bulk elemental silicon component (light blue shaded) and to the de-oxidized surface, where the restatom (RA) surface states (at lower binding energy than the bulk line, dark blue shaded) are clearly apparent. Intermediate panels are spectra measured in time resolved mode (the so-called snapshot mode, the spectra are corrected from detector distortions) along with their corresponding spectral reconstruction. At 1067 K the component shifted by −0.9 eV from the main elemental Si component (dark green shaded) is due to two contributions, the Si^1+^ oxidation state, and a clean surface state (see text). Note the increase in RA intensity from 1110 K to 1200 K (7×7 to “1×1” transition).

For the two different $\,{v}_{av}$, the main features are similar: the oxide state components at lower kinetic energies suddenly disappear during the heating process. At the same time the surface component related to the restatoms (RA) of the clean surface, indicated by a blue arrow in Fig. 2(d), appears at higher kinetic energy than the bulk peak.

**Figure 4:**
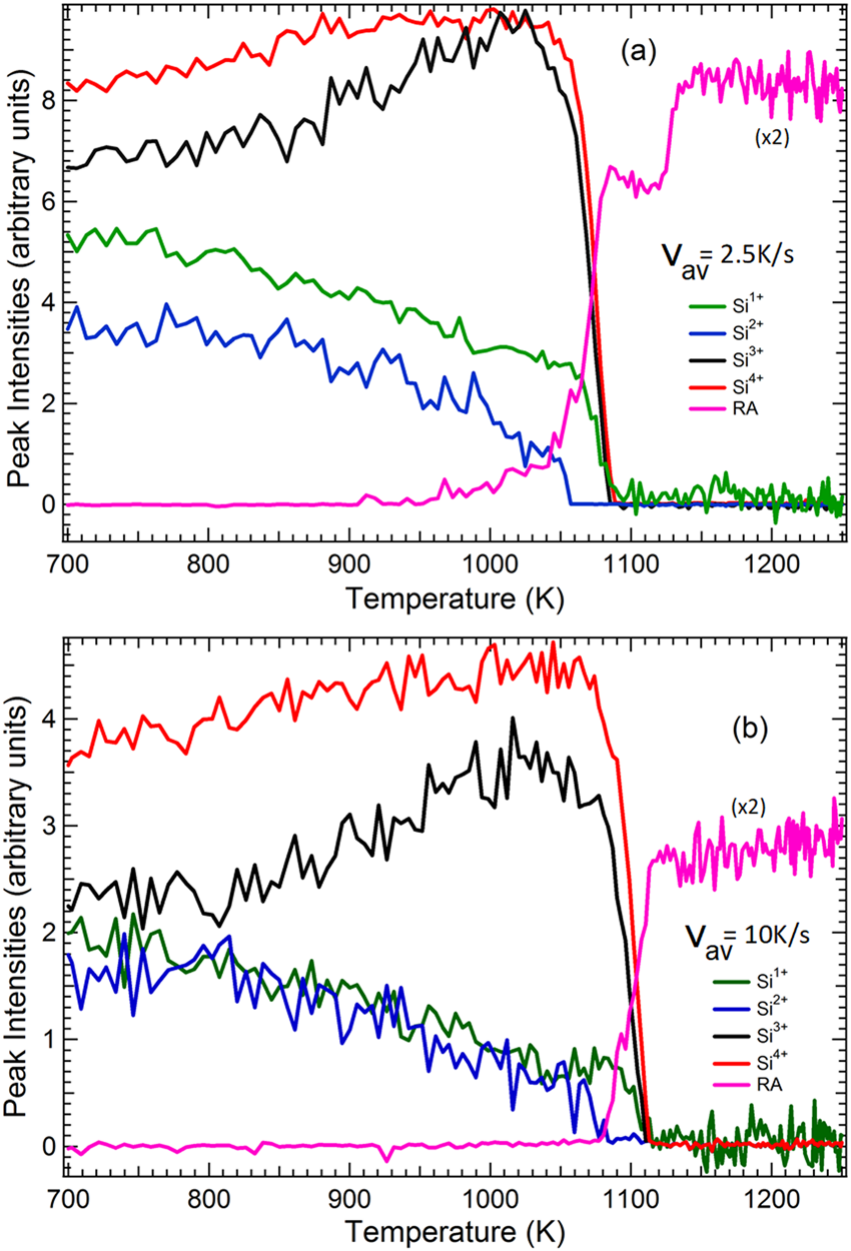
Temperature dependence of the Si oxide and suboxide intensities and of the RA contribution (pink line) for the temperature schedules (**a**) *v*_*av*_ = 2.5 K.s^−1^ and (**b**) *v*_*av*_ = 10 K.s^−1^. Note that the Si^1+^ intensity is set to zero after the desorption event due to an energy overlap with an elemental silicon component of the cleaned surface (see text).

For the slow heating schedule of 2.5 K.s^−1^, four selected Si 2p spectra measured in the “snapshot mode” at significant times indicated by red vertical lines in Fig. 2 are reported in Fig. 3. Each spectrum is integrated over a time period of 250 ms. The detector response is now taken into account, after calculating the ratio between a spectrum measured in the sweep mode and one measured in the snapshot mode. For the sake of comparison, we add two spectra measured in the sweep mode at room temperature, one of the oxygen covered surface and one of the cleaned one. Detailed information on the fitting procedures is given in the SI, section S4. For the oxidized surface, the four oxidation states $S{i}^{n+}$, where *n* = {1, 2, 3, 4} is the number of oxygen ligands around the Si atom, are seen, shifted to higher binding energy from the elemental silicon line Si^0^ by ~+0.9 eV per O ligand^40–42,52^. For the oxidized interfaces, the main line *Si* ^*0*^ is accompanied by two satellite lines whose relative binding energy is shifted by −0.4 and +0.3 eV^53^. For the cleaned surface, the present instrumental resolution (125 meV) does not make it possible to reveal the Si 2p fine structure reported and discussed in refs^43,54^. Notwithstanding, we can clearly distinguish the restatom component (RA) shifted to lower binding from the main bulk line Si^0^ by ~−0.7 eV^40–42^. Other minor components at about −0.3, +0.3 and +0.9 eV from Si^0^ must be introduced in the fitting procedure. The latter one (maybe a shakeup^43,55^), overlaps in energy with the Si^1+^ state of the oxidized surface (see Fig. 2, and more especially the 1067 K spectrum where both oxidized and cleaned areas coexist). This leads to a vertical, constant offset in the Si^1+^ intensity after the surface is fully cleaned. Consequently, in Fig. 4, the Si^1+^ intensity is set to zero after the desorption event.

**Figure 5:**
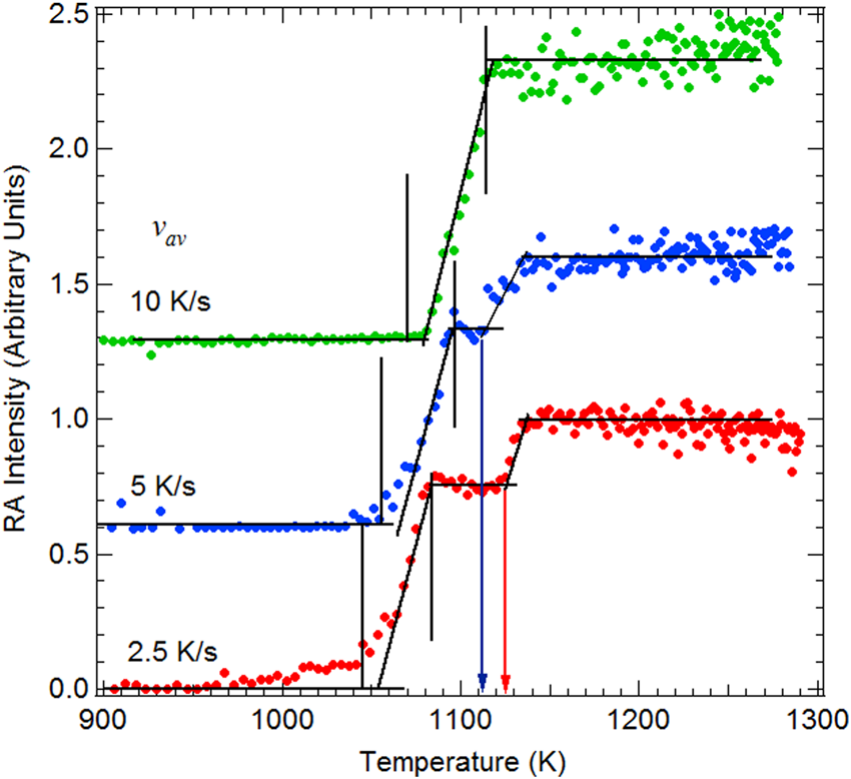
RA intensity versus temperature curves, for three temperature schedules. The curves are given a vertical offset for the sake of clarity. The desorption temperature intervals (95-5% of the Si^4+^ intensity) are indicated by the vertical lines. The 7 × 7 DAS to “1 × 1” transition temperature is indicated by a red (blue) arrow for ${v}_{av}$ equal to 2.5 Ks^−1^ (5 Ks^−1^).

**Figure 6:**
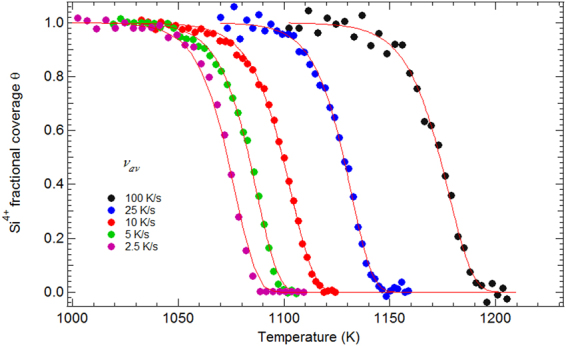
Dependence of the Si^4+^ fractional coverage θ (squares) with temperature for five different heating schedules with average velocities ${v}_{av}$. Red solid lines are fits obtained with the 3-parameter Avrami model. Corresponding parameters are given in Table 1 together with the χ^2^.

The temperature dependence of the $S{i}^{n+}\,$intensities, along with that of the RA component is reported in Fig. 4 as a function of temperature, for two illustrative heating schedules, the slow one (*v*_*av*_ = 2.5 K.s^−1^) reported in panel (**a**) and a four times faster one (*v*_*av*_ = 10 K.s^−1^) reported in panel (**b**). Specific temperature curves of the RA and Si^4+^ components for the various ${v}_{av}$ are reported in Figs 5 and 6, respectively. The “desorption temperature interval” defined by the 95-5% intensity of the Si^4+^ component is given in Table 1.

**Table 1:** The desorption temperature interval is comprised between 95% and 5% of the Si^4+^ component intensity (Fig. 6).

*v*_*av*_ (K.s^−1^)	*desorption interval 95-5% Si* ^*4+*^ *(K)*	*T*_*peak*_ (K)	*v*_*peak*_ (K.s^−1^)	*n*	*E*_eff_ (eV)	*k*_0_ (s^−1^)	*χ* ^*2*^ *(*×*10* ^−*3*^ *)*
2.5	1049−1084	1073.6	2.6	2.148	4.125	2.42 × 10^18^	5.166
5	1057−1098	1087.4	4.7	2.151	4.060	1.44 × 10^18^	10.11
10	1071−1115	1103.3	8.8	2.017	4.061	1.43 × 10^18^	7.5
25	1102−1143	1131.5	20.6	2.200	4.200	4.69 × 10^18^	20.78
100	1144−1190	1177.7	91.6	2.061	4.203	3.75 × 10^18^	27.24
Average	—	—	—	2.115	4.13	2.75 × 10^18^	—

The temperature of maximum desorption rate (T_peak_) corresponds to a fractional coverage equal to $\frac{1}{{\rm{e}}}=0.368$ ^38^ $.$ ${{\rm{v}}}_{{\rm{peak}}}\,$is the heating velocity at T_peak_. Avrami exponent (n), coefficient (k_0_) and effective activation energy (E_eff_) are deduced from the fits of Fig. 6. The fitting method is described in “Methods”, subsection “Fitting of the desorption curves with a non-isothermal Avrami Kinetic Model”. The Chi-square value (χ^2^) is also given.

Concerning the former point, different reaction channels, with different activation energies, can be explored when high velocity ramps are applied, as kinetics dominates thermodynamics. When the 7 × 7 reconstructed surface is oxidized at room temperature, as is the case here, one obtains a relatively even distribution of suboxides, with a sizeable weight for the metastable Si^2+^ component^42,56^. While for very slow temperature ramps, quasi-equilibrium conditions prevail and the system necessarily passes through the thermal interface suboxide distribution (with predominant Si^3+^ and Si^1+^ states^42,56^), very fast temperature ramps may not completely deplete the metastable interface states Si^2+^, modifying the decomposition process. Similarly, high heating rates could affect the surface reconstruction of the cleaned areas (and we shall see it is indeed the case). Therefore, spectroscopic information on the suboxide distribution and on the surface state intensities (in the clean areas) is decisive for understanding the desorption process.

Let us first focus on the behavior of the Si suboxides states with temperature. For the slow heating schedule of *v*_*av*_ = 2.5 K.s^−1^, see Fig. 4(a), the desorption temperature interval is 1049−1084 K. However, modifications in the oxide state distribution are already seen from ~850 K. While the *Si* ^4+^ state intensity increases slowly, the increase in the *Si* ^3+^ state intensity is much steeper. The *Si* ^1+^ state decreases continuously until the desorption event. In contrast with the other suboxide states *Si* ^3+^ and *Si* ^1+^, the temperature behavior of the *Si* ^2+^ component is different. It decreases continuously and becomes non-measurable at the onset of oxide desorption (1050 K), that is ~30 K before the surface is fully cleaned from its oxide (when the *Si* ^4+^ intensity is zero).

For a faster heating schedule of *v*_*av*_ = 10 K.s^−1^, Fig. 4(b), the desorption temperature interval is shifted to higher temperature (1071−1115 K). As expected for activated processes, kinetic events are function of both time and temperature: the transition shifts to higher temperature when heated at a higher rate, because of the reduced time spent at each temperature. The behaviors of the four Si oxidation states are similar to those observed at a slower ${v}_{av}$ of 2.5 K.s^−1^. The Si^2+^ signal becomes unmeasurable at 1081 K, that is ~34 K before the Si^4+^ intensity goes to zero, as observed for the slower heating schedule.

The same type of Si/SiO_2_ interface, with dominating Si^3+^ and Si^1+^ suboxide states, is formed for the two different heating schedules, thus the chemistry of the Si/SiO_2_ interface seems independent of the thermal history. After O_2_ exposure at room temperature, the dimer-adatom-stacking fault (DAS) structure is decorated by O atoms inserted within the silicon-silicon bonds. Upon heating, the oxidized DAS is destroyed, and the characteristic thermal SiO_2_/Si(111) interface forms^42^. *Si* ^*1+*^ and *Si* ^*3+*^ states are preponderant, simply because of the orientation of the surface (in a diamond cubic lattice, a cut of the (111) plane give surface atoms exhibiting only one or three dangling bonds). Oxygen atoms migrate at the surface, but do not leave it at temperatures lower than the Si^4+^ desorption front, as shown by the O 1 s intensity curve versus *T* plotted in the supporting information, section S3, Figure S3.

If we now concentrate on the RA intensity curve (pink line in Fig. 4) of the slow heating schedule of 2.5 K.s^−1^, we observe it starts to increase at ~1000 K (Fig. 4(a)), that is ~50 K before the desorption interval, as defined before (5–95% of Si^4+^ intensity). This means that the suboxide rearrangement leaves clean silicon patches that make possible the appearance of the tri-coordinated adatoms (AD) and of RA, and charge transfer from the AD (in excess with respect to RA) to the RA dangling bonds, that become fully occupied^57^. This is a clear proof of the formation of de-oxidized areas before the SiO_g_ desorption event. These areas are likely the nuclei from which the desorption process (depicted in Fig. 1) will start. In this regime, the nuclei are already formed before the transformation. Once oxygen atoms start to migrate away, a corner hole opens due to AD movements, as depicted in the work of Ohdomari and coworkers^58^, leading to the DAS reconstruction. With the present slow heating schedule, the 7 × 7 DAS structure has enough time to form. Naturally the RA intensity shows a steep increase at the desorption edge, where the oxide state intensities suddenly fall. When we consider now the faster (10 K.s^−1^) schedule (Fig. 4(b)), we observe that the RA signal starts to take off within the desorption interval. Due to the high temperature rate the system may have not enough time to produce well-ordered 7 × 7 DAS areas before the desorption event.

After the desorption event, the behavior of the RA intensity as a function of temperature is illustrated in Fig. 5 for three different heating. Very interestingly, a second rise in intensity of the RA is seen at about 1128 K (midpoint) for the slower ramp with *v*_*av*_ = 2.5 Ks^−1^. This point was also clearly visible in the 3D map of Fig. 2(b) and in the spectra shown in Fig. 3 (compare the 1110 K and 1200 K spectra). The temperature of 1130 K is precisely that of the 7 × 7 DAS → “1 × 1” phase transition^47,48^, above which an increase in the RA intensity was observed by Paggel *et al*. in the Si 2p spectrum^59^. These authors proposed that the so-called “1 × 1” is in fact a disordered 2 × 2 reconstruction on top of an ideal 1×1 bilayer of the bulk-terminated Si(111) surface, presenting one RA and one AD per unit cell. The electron in the AD dangling bond can be transferred to the RA one that becomes doubly occupied, and thus negatively charged, which also leads to a surface core-level state at lower binding energy than that of the bulk component. The RA coverage of the 2 × 2 reconstruction is $\frac{1}{4}$ ML (0.25 ML), higher than that of the 7 × 7 DAS that amounts to $\frac{7}{49}$ ML (0.14 ML), and therefore the increase in the surface state intensity can be understood. Note that the non-linear optical spectroscopy study by Höfer *et al*.^60^ indicates a substantial increase in the dangling bond surface density above the transition temperature.

The remarkable point is the fact that the 7 × 7 DAS to “1 × 1” transition temperature changes with the ramp velocity (Fig. 5). For the 5 K.s^−1^ schedule, the RA intensity jump at the 7 × 7 DAS to “1 × 1” transition is still visible, but at a distinctly smaller value of ~1120 K (midpoint). For an activated kinetic process, involving dimer breaking and elimination of the DAS, the opposite would be expected. Our result suggests that the clean areas of the *v*_*av*_ = 10 K.s^−1^ ramp are composed of less ordered 7 × 7 DAS surface cells that transit to “1 × 1” below their expected thermodynamic phase transition temperature of ~1130 K. The formation of strained, smaller DAS cells^61–63^ can be equally envisaged. Note that the RA signal takes off at 1050 K, which corresponds to the onset of the Si^4+^ decay, see Fig. 6, which contrasts with the 2.5 K.s^−1^ ramp for which the nucleation of clean areas with significant RA intensity predated the desorption process.

For the ${v}_{av}\ge 10\,{\rm{K}}.{{\rm{s}}}^{-1}$ ramps, the RA intensity curves do not present the characteristic jump of the 7 × 7 to “1 × 1” phase transition. The RA intensity of the $10\,{\rm{K}}.{{\rm{s}}}^{-1}$ ramp saturates at 1120 K (when the Si^4+^ intensity is zero). For the faster schedules the surface becomes clean at temperatures of 1150 K (25 K.s^−1^) and 1190 K (100 K.s^−1^), that are significantly above the 7 × 7 to “1 × 1” transition temperature. As the 7 × 7 DAS to “1 × 1” transition temperature decreases below the “thermodynamic” one when the temperature rate increases, there is no reason to believe that the 7 × 7 is formed in this case.

This spectroscopic information gives clues on the nucleation of the clean areas and on their reconstruction. The faster the ramp the more disordered (or strained) the de-oxidized patches, and the 7 × 7 DAS is no more an intermediate. The question that arises here is whether this has an impact on the kinetic parameters of oxide desorption, as the Avrami exponent and effective activation energy depend on the nucleation process.

### Kinetic analysis

Time-resolved Si 2p spectroscopy gives clear evidence that oxygen rearrangement and elimination of the DAS structure leads to a characteristic SiO_x_ interface prior to de-oxidation. During the desorption process the *spectroscopic* data indicate that the *Si* ^*4+*^, *Si* ^*3+*^ and *Si* ^*1+*^ oxide disappear at the same pace. Naturally this can be accounted for by the growth of clean silicon voids at the expense of the ultra-thin thermal oxide (Fig. 1). Consequently, the most intense Si^4+^ component is used as a “proxy” to extract the kinetic parameters of the oxide desorption process.

In Fig. 6 we plot the Si^4+^ fractional coverage as a function of temperature for the five different temperature schedules (see Methods, subsection “Real-time Photoemission”). The temperature of the maximum desorption rate (${T}_{peak}$) reached at a fractional coverage $\theta$ of ~$1/e$ (0.37)^38^ is reported in Table 1 as well as the corresponding temperature velocity ${v}_{peak}$ (calculated using eq. 8 in the SI). The desorption fronts (midpoints) shift to higher values for increasing heating velocities by about 110 K (from ~1070 to ~1180 K). This vast temperature extension warrants a good evaluation of the robustness of the Avrami modelling and the stability of the kinetic parameters over the 7 × 7 to “1 × 1” thermodynamic transition temperature of ~1128 K as discussed before. The desorption fronts are fitted by the mathematical formulae given in “Methods”, subsection “Fitting of the desorption curves with a non-isothermal Avrami kinetic model” (Eqs (5) and (6)), that are an adaptation of the Avrami formalism to the present non-isothermal case where the heating schedules are not linear with time. The fitting parameters are the Avrami exponent *n*, on the one hand, and the effective activation energy ${E}_{eff}$ and the pre-exponential factor ${k}_{0}$ (a frequency) of the Avrami coefficient $k$, on the other hand (see “Methods”, subsection “Fitting of the desorption curves with a non-isothermal Avrami kinetic model”). All three parameters were let free. We observed that the value of *n* shapes the desorption fronts. For instance, by forcing *n* to 1 (see SI, section S5, Figure S5), we could not fit properly the steepness of the desorption front. Indeed, a first-order reaction accounts for a homogeneous system, which is definitely not the case here^31^. The fitting values are collected in Table 1.

Table 1 shows that *n* close to 2 (the average is 2.1) gives excellent fits of the desorption steps for all the heating schedules (the increase in *χ* ^2^ observed for the faster temperature programs is due to the “noisy” Si^4+^ intensities and not to the fitting accuracy), showing that the Avrami modelling is robust through a wide ${T}_{peak}$ range. For a 2D system, an *n* value of 2 is indicative of “simultaneous nucleation” (see “Methods”, subsection “Fitting of the desorption curves with a non-isothermal Avrami kinetic model”) i.e. desorption nuclei (i.e. clean surface area) preexist (as shown by the RA intensity versus T curve of the 2.5 K.s^−1^ ramp in Fig. 4), or form immediately on the surface when oxygen desorbs. In such a case $\,{k}_{0}\,\,$is proportional to $\sqrt{N}$ where $\,N$ is the density of nuclei, and the activation energy ${E}_{eff}$ is simply ${E}_{G}$, that of the void growth velocity ${v}_{G}$. We find that ${E}_{eff}\,($=*E*_*G*_) is 4.1 ± 0.1 eV. The Avrami model that takes explicitly into account the spatial non-homogeneity of the desorption process, gives a single-valued activation energies, in contrast with the coverage-dependent activation energies of a Taylor-Weinberg analysis^2^. This value is good accord with void growth rate activation energies obtained via microscopy measurements^13,17^ for oxidized Si(001), that are all close to ~4.0 eV, in a vast range of oxide film thicknesses, between ~1 and 120 nm. Experimental and theoretical data concern only step 4, in the limit case of “zero coverage”. The experimental activation barrier for SiO_g_ desorption on Si(111) has an upper bound value of 4.4 ± 0.15 eV (Memmert *et al*.^3^) and a lower bound one of 3.3 eV (Engstrom *et al*.^2^), the latter one being close to what is found for Si(001)^1,2,5,6^. Desorption energies were not calculated for the Si(111) surface. The calculated desorption barrier of a single oxygen atom “embedded” in the otherwise clean silicon (001) surface (no oxygen clustering is considered), is in the 3.5–3.9 eV range (3.53–3.87 eV in a quantum mechanical calculation^19,21^ and 3.60-3.80 eV in a plane wave density functional theory calculation^18^).

The stability of the kinetic parameters ${k}_{0}\,\,$and ${E}_{eff}$ is remarkable and is a valuable clue to understanding the nucleation process. This means that *N* is about the same for the slow heating schedules and for the faster ones, despite the differences in RA peak appearance. ${E}_{eff}$ (=${E}_{G}$) is also insensitive to the desorption front (${T}_{peak}$) occurring below or above the 7 × 7 to “1 × 1” phase transition at 1130 K. The nature of the surface reconstruction is unimportant, suggesting that step 1 (adatom formation) and step 2 (adatom diffusion) are not limiting steps. Only step 3 (formation of precursor) or step 4 (SiO_g_ desorption) matter.

Finally, our fitting procedure leading to the extraction of the three kinetic parameters differs from the Avrami analysis by Kinefuchi and coworkers^7^ who chose deliberately an activation energy equal to that of SiO_g_ desorption at nearly zero oxygen coverage^6^. This *a priori* choice may lead to a non-integer *n* exponent between 2 (instantaneous nucleation) and 3 (constant nucleation rate), of unphysical meaning (see “Methods” subsection “Fitting of the desorption curves with a non-isothermal Avrami kinetic model”), unless one concedes it reflects a nucleation rate that decays exponentially with time^64^. Moreover the latter authors used a single heating program (that is sufficient in principle), but did not explored the range of application of their modelling by varying the heating velocities and thus the desorption peak temperatures.

## Concluding remarks

We have used thermally programed XPS (TPXPS) to monitor the de-oxidation process of the 7 × 7 DAS Si(111) oxidized at room temperature. The combination of the synchrotron radiation high flux and a fast acquisition system synchronized to the temperature measurement and to a controlled heating program, lead to the recording of “chemically meaningful” spectra with a time resolution of 50 ms. The maximum heating velocities we have reached are now competitive with TPD experiments based on mass analysis, and are also comparable to the temperature rise of rapid thermal annealing processes, commonly used in semiconductor surface processing, which warrants a wide range of applications for TPXPS. With respect to previous thermally programed desorption (TPD) experiments that monitor the SiO_g_ desorption rates, fast (*ms*) Si 2p spectroscopy gives access to changes in the distribution of the oxidation states, before and during the SiO_g_ desorption event, to which TPD is obviously blind. TPXPS adds also chemical evidence to the desorption models that microscopies have provided, emphasizing the complementarity of the two approaches.

TPXPS sheds new light on the formation of clean areas by monitoring the intensity of the restatom component. We see that the temperature of the phase transition 7 × 7 DAS to disordered “1 × 1” reconstruction decreases when the temperature velocity increases. Defective 7 × 7 DAS cells (or maybe smaller DAS cells) might be formed, with a lower transition temperature to the “1 × 1”.

Avrami’s nucleation-growth model is implemented through the derivation of mathematical expressions that account for the non-linearity of the heating schedules. This enables us to extract the kinetic parameters (a triplet) that describe the de-oxidation process. The Avrami exponent is close to 2, pointing to a 2D process for which the nucleation of clean areas is instantaneous. The clean area growth activation energy is of ~4.1 eV. The question remains as to whether the limiting step is the desorption of SiO_g_ into the gas phase, or the formation of a species precursor to desorption by reaction of Si with SiO_2_. Hopefully, this work will stimulate experimental and theoretical studies on the reaction of silicon monomers with the surface of silica. The wide range of heating velocities translates into a ~110 K temperature window for the desorption fronts (encompassing the static 7 × 7 DAS to disordered 1 × 1 transition temperature), within which the fitting parameters are found very stable. The present analysis can find useful applications when desorption of the oxide layer is a fundamental step in nanofabrication processes on silicon surfaces.

## Methods

### Real-time photoemission

Real-time desorption experiments were performed in the UHV photoemission experimental station of the TEMPO beamline at the SOLEIL synchrotron radiation facility^65^. For the Si 2p spectra (of binding energy ~100 eV) the choice of the photon energy is conditioned by the pass energy (PE) that defines the “snapshot” energy window width (6% of the PE). A width of 6 eV is necessary to encompass the oxidation states, and the elemental silicon components. Therefore, a PE of 100 V was used. As the Scienta SES200 works correctly with photoelectrons of kinetic energies greater than PE, we used a photon energy hν of 210 eV to record the Si 2p snapshot window. The inelastic mean free path λ of photoelectrons of kinetic energy ~110 eV in Si is 5 Å^40^. As the takeoff angle of the photoelectrons was 45° from normal, the effective escape depth, λ $\times \,\cos (45^\circ )$, is 3.5 Å. This value is comparable to the minimum λ, 3.3 Å, ref.^40^ obtained when the kinetic energy is ~30 eV, i.e. when hν = 130 eV. The overall instrumental resolution was ~125 meV. The Si 2p core-level is actually a doublet with a spin-orbit splitting of 0.61 eV and a 2p_3/2_:2p_1/2_ branching ratio of 2. For their part, O 1 s spectra were measured at a photon energy of 640 eV (see supporting information, section S2).

The (111)-oriented silicon substrates (of dimensions 5 mm × 10 mm × 0.25 mm) were phosphorus-doped (resistivity 10^−3^ Ω.cm), thoroughly degassed overnight at 600 °C and cleaned from their native oxide by Joule heating before exposure to dry oxygen in controlled conditions. To obtain spatially homogeneous and reproducible starting oxygen coverage, the formation of thermal oxide by Joule heating in the presence of O_2_ was not considered as practicable because of temperature gradient across the sample and difficulties in keeping the temperature fixed with time. Therefore, the surface oxidation was carried out at *room temperature* under a pressure of ~5 × 10^−8^ mbar. As in the very initial regime faulted half-cells are the more reactive than unfaulted ones, a homogeneous coverage on both half-cells is obtained at room temperature for O_2_ doses of ~45 L (1 L = 10^−6^ Torr × s), see ref.^66^ and its supporting information. At such high doses, the oxygen uptake is also very slow, as shown by the adsorption kinetic curve given in Figure S2(a) of the SI. An estimated O coverage of 1.4 ML (1 ML = 7.68 × 10^14^ atoms/cm^2^) was obtained. The O 1 s spectrum at 45 L (Figure S2(b) of the SI) shows clearly the absence of molecular metastable oxygen^67,68^, observed at much lower exposure (~1 L).

To extract significant kinetic parameters relative to the thermal de-oxidation process, several heating schedules with average ramp velocities ${v}_{av}$ between ~2.5 K/s and ~100 K/s were applied to the oxygen covered wafer. Therefore, it was crucial to start with identical surface conditions. In particular, the sample surface must not roughen after repeated oxidation/de-oxidation cycles. This was ensured by (111) oriented wafers: this orientation is stable with respect to step formation during oxide sublimation^69^, in contrast to the (001) orientation that can be heavily etched^1^. The room temperature oxidized Si wafers were heated by direct current injection in front of the electron analyzer while performing photoemission experiments. Reproducible and scalable heating rates were performed using predefined voltage shapes from a Tektronix programmable function generator driving a KEPCO power supply [200 W BOP-E] operated as voltage to current amplifier. The temperature was measured for each experiment using a fast response pyrometer “IMPAC IS^−1^2 Si” dedicated to silicon surface measurements (emissivity  = 0.67). The “IMPAC IS^−1^2 Si” can only measure temperatures from 600 K.

As Joule heating induces a potential drop dependent on the sample resistance, and hence a shift in the measured kinetic energy of the core level spectrum, we submitted the silicon sample to a “comb-like” voltage ramp, with current pulses of 25 ms and zero bias intervals of 25 ms. High resolution photoemission spectra are obtained at zero bias: the sample is grounded and the Fermi level is the same as that of the analyzer.

The voltage comb was synchronized with the photoemission data acquisition to separate signals obtained during current flow and zero applied voltage. We used the fast acquisition system of the TEMPO beamline^39^ set at 25 ms/spectrum, corresponding to a snapshot periodicity of 50 ms (measurement at zero bias).

The resulting temperature ramps $T(t)$ were non-linear in the regions of interest (e.g. the desorption event), see the yellow curves in Fig. 2. We fitted the useful part of temperature curve with a function of the type:

1 $$T({t})={{T}}_{{\boldsymbol{m}}}(1-\exp (-\frac{{\rm{\phi }}}{{{T}}_{{\boldsymbol{m}}}}{t}))$$

where *T* < *T*_*m*_.

The parameters $\phi$ and ${T}_{m}$ are given in Table 2. Temperature ramps of duration $\,{\tau }_{ramp}$ are denominated according to their average velocity ${v}_{av}$.

**Table 2:** Parameters (${{\rm{T}}}_{{\rm{m}}}$ and ${\rm{\phi }}$) of the heating schedules of duration ${{\rm{\tau }}}_{{\rm{ramp}}}$ and average velocity ${{\rm{v}}}_{{\rm{av}}}$.

$\,{{\boldsymbol{\tau }}}_{{\boldsymbol{ramp}}}$ **(s)**	${{\boldsymbol{v}}}_{{\boldsymbol{av}}}$ **(K.s** ^**−1**^ **)**	***T***_**m**_ **(K)**	***φ*** **(K.s** ^**−1**^ **)**
400	2.5	1295.4	15.3
200	5	1293.9	29.68
100	10	1278.2	64.64
40	25	1293	164.86
10	100	1324.9	825.28

The non-constant temperature rates preclude any precise application of classic “Arrhenian” methods such as Kissinger plots^38,70^. For non-isothermal transformations, Kissinger plots present also the disadvantage of providing only the activation energy, as the determination of the Avrami exponent is cumbersome, and finally inaccurate^38^.

### Fitting of the desorption curves with a non-isothermal Avrami kinetic model

In the Avrami formalism, nuclei are distributed at random and the nucleation and growth laws are given *a priori*. In the isothermal case, the fraction of the untransformed phase *θ* is given by the simple formula^34–38^:

2 $${\theta }={{e}}^{-{{\alpha }}_{{ex}}}$$

where ${\alpha }_{ex}$ is the so-called extended transformed phase fraction, that is the fractional surface the transformed phase would acquire if the overlap among the growing nuclei were disregarded. For simultaneous or continuous nucleation and provided the growth law is a time power, α_ex_is expressed as a function of the transformation path $\beta =kt$ function of under the form:

3 $${\alpha }_{ex}={\beta }^{n}={(kt)}^{n}$$

where t, k and n are the running time, the Avrami coefficient (a frequency) and the Avrami exponent (an integer), respectively.

Considering the 2D case, the limiting step is the reaction occurring at the periphery of the clean (circular) areas, and hence the rate is proportional to the radius. By assuming a *constant* growth rate for the transformed phase (${v}_{G}$ is the void radius growth velocity) and simultaneous nucleation ($N$, the surface density of nuclei is constant), then ${\alpha }_{ex}=N\pi {({v}_{G}t)}^{2}$. In such a case, the Avrami exponent *n* is equal to 2 and the Avrami coefficient $k$ is simply

4 $$k=\sqrt{\pi N\,}{v}_{G}$$

When ${v}_{G}$ remains constant, but the density of nuclei increases linearly with $t$, it can be easily shown that the Avrami exponent *n* is equal to 3 (see section S5 of the SI).

In the case of non-isothermal experiment, we calculate a transformation path $(T)$ (depending on the temperature T) that substitutes that of the isothermal transformation. We assume that the Avrami coefficient *k* follows an Arrhenian dependence of the type $k={k}_{0}\,\exp (-{E}_{eff}/{k}_{B}T$). Then one obtains (see SI section S5 for the detailed calculation):

5 $${\rm{\beta }}({T})=\frac{{{k}}_{0}{{T}}_{{\boldsymbol{m}}}}{{\rm{\phi }}}\{-{\exp }(-\frac{{{E}}_{{\boldsymbol{eff}}}}{{{k}}_{{B}}{{T}}_{{m}}}){Ei}(-\frac{{{E}}_{{\boldsymbol{eff}}}}{{{k}}_{{B}}}(\frac{1}{{T}}-\frac{1}{{{T}}_{{m}}}))+Ei(-\frac{{E}_{eff}}{{k}_{B}T})\}$$

where ${T}_{m}$ (K) and $\phi$ (K.s^−1^) are the fitting parameters of the non-linear temperature schedules reported in Table 2. Ei is the exponential integral ${\rm{Ei}}(x)=-{\int }_{-{\boldsymbol{x}}}^{\infty }\frac{{{e}}^{-{\boldsymbol{t}}}}{t}dt$.

The fraction of untransformed matter (*T*) is simply written as:

6 $$\theta (T)=\exp (-{[{\rm{\beta }}(T)]}^{{\boldsymbol{n}}})$$

In the fitting procedure of the transformation steps φ and ${T}_{m}$ are fixed parameters. The kinetic parameters *n* (the Avrami exponent), $k{}_{0}\,\,$the pre-exponential factor of the Avrami coefficient and $E{}_{eff}$ the effective activation energy are let free. With a growth velocity ${v}_{G}={v}_{G,0}\exp (-\frac{{E}_{G}}{{k}_{B}T})$, where ${v}_{G,0}$ is the growth velocity pre-exponential factor, and for instantaneous nucleation, $n$ = 2, ${k}_{0}=\sqrt{\pi N}{v}_{G,0}$ and ${E}_{eff}={E}_{G}$.

### Data Availability

The datasets generated during and/or analyzed during the current study are available from the corresponding author on reasonable request.

## Electronic supplementary material

Supporting Information
