# Supplementary material for: Chemical and kinetic insights into the Thermal Decomposition of an Oxide Layer on Si(111) from Millisecond Photoelectron Spectroscopy
Source: Sci Rep. 2017 Oct 27;7:14257. doi: 10.1038/s41598-017-14532-4 (PMC5660199; doi:10.1038/s41598-017-14532-4)
Supplement: Supplementary file 1 — Supporting Information [file 41598_2017_14532_MOESM1_ESM.pdf]

SUPPORTING INFORMATION

Chemical and kinetic insights into the Thermal Decomposition of an Oxide Layer on Si(111) from Millisecond Photoelectron Spectroscopy

J.-J. GALLET,[1,2] M. G. SILLY,[2] M. EL KAZZI,[2,†] F. BOURNEL,[1,2] F. SIROTTI,[2,3] F. ROCHET.[1,2,*]

[1] *Sorbonne Universités, UPMC Univ. Paris 06, UMR 7614, Laboratoire de Chimie Physique Matière et Rayonnement (LCPMR), F-75005 Paris, France.*

[2] *Synchrotron SOLEIL, L'Orme des Merisiers, Saint-Aubin, BP 48, F-91192 Gif-sur-Yvette, France.*

[3]*Laboratoire de Physique de la Matière Condensée, CNRS and Ecole Polytechnique, Université Paris Saclay, F- 91128 Palaiseau, France*

[†] *Now at Paul Scherrer Institut, 5232 Villigen-PSI, Switzerland*

[*] *Corresponding author: francois.rochet@upmc.fr*

S1. Synchronized time resolved acquisition.

Photoelectron experiments are performed with a SCIENTA SES 2002 electron energy analyzer whose CCD camera was replaced by a delay line detector (DLD).[1] It allows us to determine the photoelectron detection time in pump/probe experiments with a time resolution ranging between 10 and 100 ns depending on the electrons kinetic energy and the selected pass energy.[1] Information on energy and momentum of the analyzed electrons is obtained via the spatial resolution of the DLD as for a CCD camera, but each photoelectron can be associated to a well-defined time interval. The timing of the data acquisition process is controlled using TTL signals defining significant time intervals for spectroscopic signal accumulation. A LABVIEW based library fully compatible with the functions of the original software driving the CCD camera was developed to handle the time interval information and to separate the measured events in time resolved spectra or detector images.

For non-periodic phenomena, the limitation is not the time resolution, but the signal statistic. Two different atomic configurations at the surface can be resolved if the signal to noise ratio in the measured spectrum is good enough to identify the significant structures.

The fastest data acquisition configuration is obtained when measurements are performed in the fixed mode ("snapshot") and the measured photoemission spectrum is collected directly on the energy axis of the 2D detection system. In this configuration the measured energy range is proportional to the pass energy. When the Si 2p peak of the oxidized surface is concerned, elemental and oxidation states are distributed over a binding energy range of the order of 6 or 7 eV. In order to get all the structures in one single image the minimum pass energy is 100 V.

We have chosen the photon energy (210 eV) in order to properly measure 100 eV kinetic energy electrons with a 100 V pass energy, i.e. with the highest possible surface sensitivity to increase the contribution of the oxide components at the surface and at the SiO_2/Si interface.

In order to avoid saturation problems, we have regulated the incoming soft x-rays photon flux to reach a count rate of about 150,000 photons/sec. Taking into account the peak to background ratio of Si 2p photoemission spectra at 210 eV photon energy we have considered 3,000 counts a minimum number needed to identify oxide contribution and to perform spectra deconvolution. This has fixed to about 25 ms the minimum significant integration time for the experiments. Improving detector count rate before saturation[2] could increase automatically the time resolution. The Si 2p XP spectra recorded in "snapshot" mode are corrected in intensity by the ratio between the clean surface spectrum obtained in sweep mode and the one recorded in snapshot mode.

A scheme of the experimental setup is given in Figure S1. The temperature ramp is obtained via Joule heating, by flowing current in a 250 μm thick silicon wafer (*n*-type, of resistivity 3×10^{-3} Ωcm) indicated by the red square at the center of Figure S1. The current is controlled using a bipolar operational amplifier (current generator in top center of figure S1) operated in current control driven by a voltage signal programmed by a TEKTRONIX AFG 3252 function generator (top right in the figure). The first output is used to produce burst signals of selectable amplitude, frequency and duration to heat the silicon wafer (on the sample holder at the center of the figure). For these experiments it was set to produce a square wave with frequency f=20 Hz (50ms period) while the voltage and the duration were calibrated to select the heating rate and to reach the desired maximum annealing temperature. This channel, with 50 ms square cycles, sets the current delivered by the KEPCO supply. Energy is transferred to the silicon sample during the 25 ms when the current flows through the silicon wafer.

During this time interval a voltage drop appears across the silicon sample due to its nonzero resistivity: the photoemission peaks are shifted in kinetic energy from their "grounded" kinetic energy value. To avoid this disturbance that hinders seriously the measurement of binding energies, the "energy calibrated photoemission spectrum is acquired during the 25ms when the applied voltage is set to zero (i.e. the sample is grounded).

The data acquisition is driven by the second channel of the function generator which is synchronized to the first one and set to a frequency $2f$ (40 Hz) with 5V amplitude to reproduce the TTL signal needed to drive the delay line detector electronics.[1] In this configuration, two photoemission spectra are measured in the 50 ms heating period: the second one is obtained when no voltage is applied to the sample and gives the energy calibrated photoemission spectra presented here.

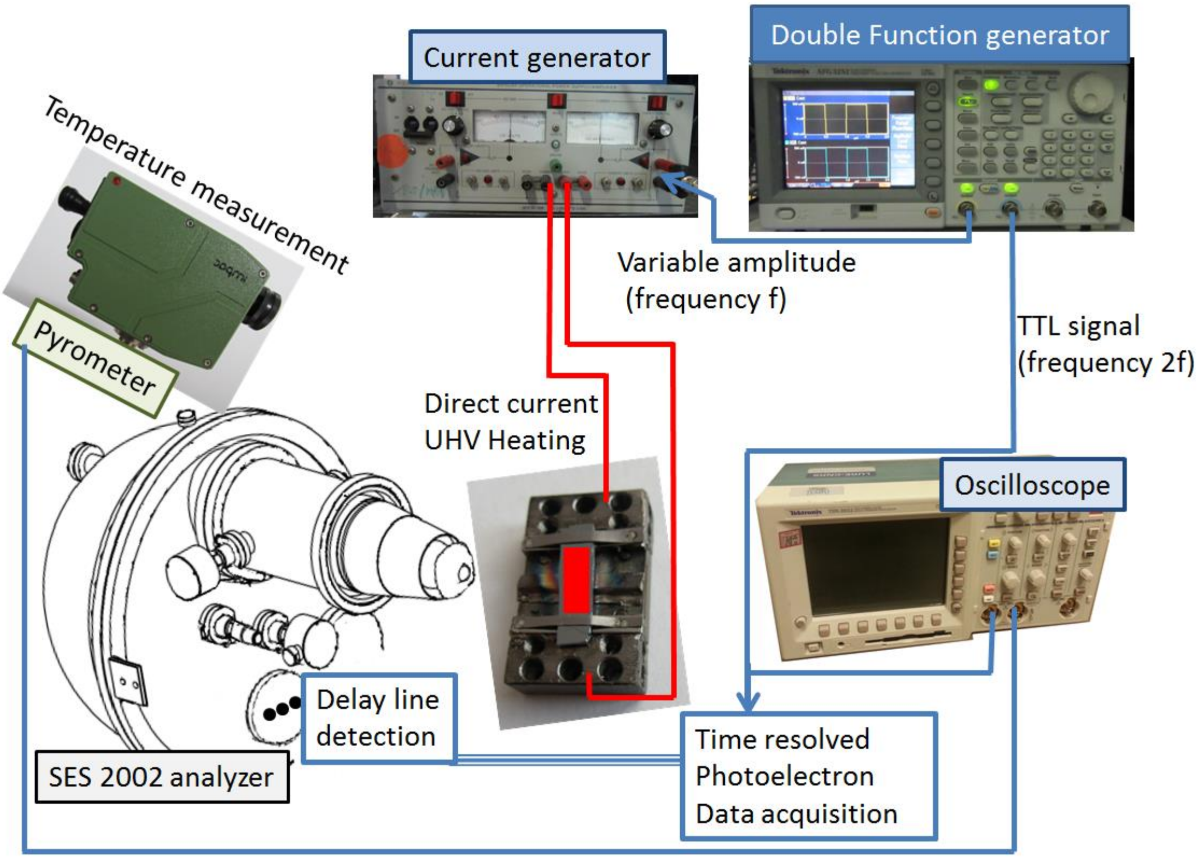

Figure S1. Scheme of the experimental setup. All photos and drawings were made by the authors.

The complete burst of the TTL signal used to control the data acquisition is measured by a 2 channels TEKTRONIX oscilloscope along with the sample temperature signal given by a fast response (<1 ms) pyrometer “IMPAC IS-12 Si” dedicated to silicon surface measurements (emissivity 0.67).

The sample temperature is directly associated to the measured spectra by the time scale of the oscilloscope. The average temperature measured by the pyrometer during the 25ms periods with zero applied voltage is associated to the corresponding photoemission spectrum.

S2. Preparation of reproducible oxide layers: oxygen dosing at room temperature

In order to perform various heating schedules on the same silicon wafer, it is essential to start from identical oxidation coverages. We have chosen to adsorb oxygen on the Si(111)-7×7 surface at room temperature, under a nominal pressure of 5×10^{-8} mbar because it is a much more reproducible procedure than growing thermal oxides. Indeed, thermal oxidation may be affected by difficulties in controlling the temperature during and the resulting oxide layer may be not homogeneous in thickness due to temperature gradients when Joule heating is used.

The “real-time” O 1s photoemission intensity plotted against O_2 dose at 300 K is presented in Figure S2 (a). Absolute coverages in ML (1 ML= 7.83×10^{14} atoms/cm^2) are given after comparison of the Auger Si LVV normalized[3] O 1s intensity with that of the Si(001) surface saturated by water (corresponding to one oxygen per Si dimer). The present calibration compares with a precise medium energy ion scattering measurement[4] giving an oxygen coverage of 1.7 ML for an exposure of 15 L. As shown in Figure S2 (a), after an initial fast uptake regime up to ~0.5 L, the O 1s intensity increases slowly. During the thermal oxygen desorption experiments, the clean surface was exposed to 45 L of O_2, corresponding to an O coverages of 1.4 ML.

The O 1s spectrum (45 L, 300 K) measured in the energy swept mode (hν=640 eV, θ_e= 46°) is shown in Figure S2 (b). It can be fitted with three Gaussians, with full-width at half maximum (FWHM) of 1.14 eV each. We find three components at binding energies of 531.73 eV (67% of the spectral weight), 532.57 eV (23%) and 533.38 eV (10%).

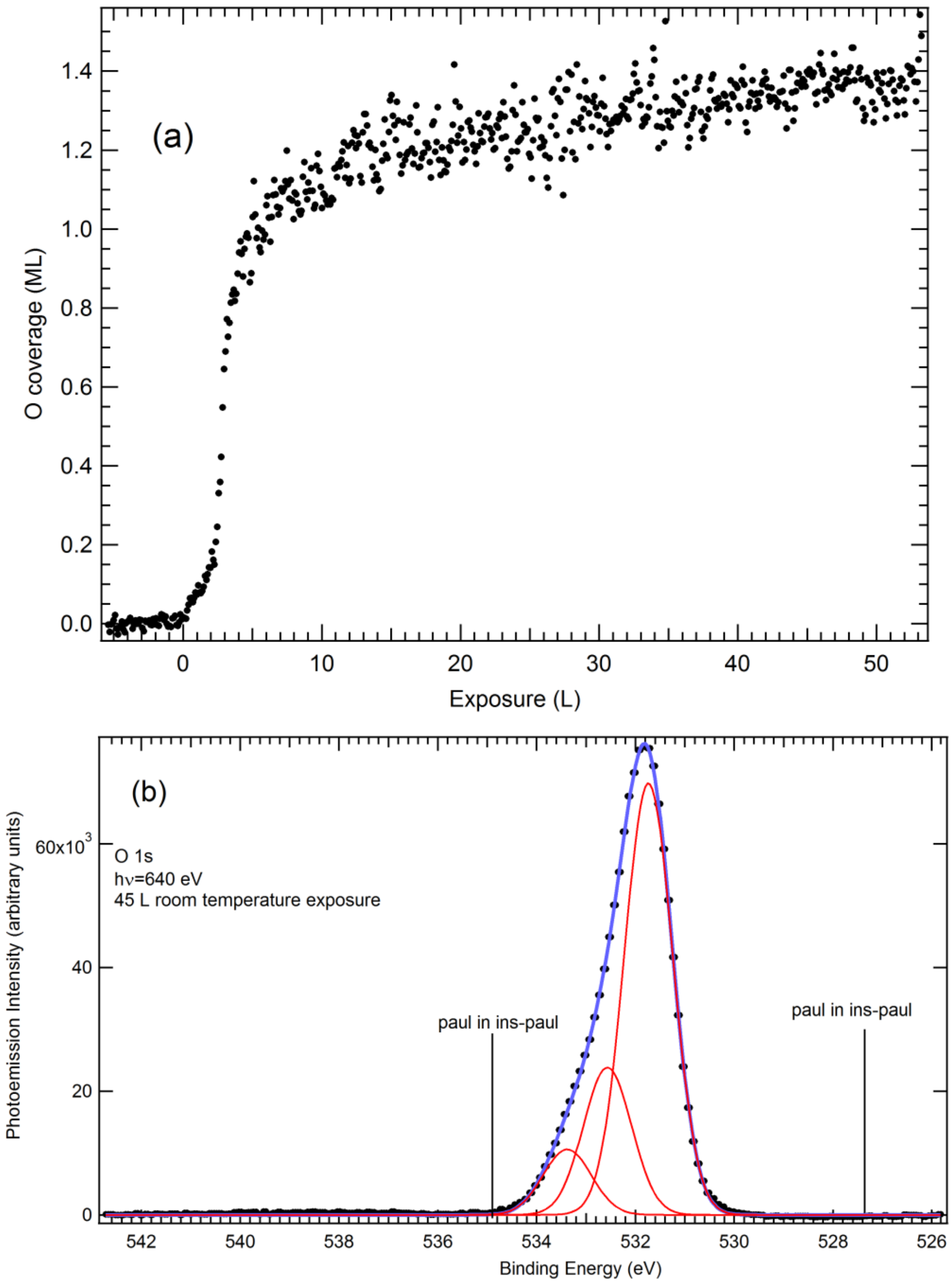

Figure S2. *(a) O 1s intensity measurement as a function of O_2 exposure (hν=640 eV) expressed in equivalent ML (for the Si(111) surface 1 ML=7.83 $\times 10^{14}$ atoms/cm^2). (b) The O 1s spectrum (45 L, exposure at 300 K) measured at a photon energy of 640 eV: dots are the experimental data after background subtraction, the red continuous curves are the fitting Gaussian lines (fwhm=1.14 eV), and the blue curve is the fitting result, sum of the three Gaussian curves.*

On the 7×7 DAS surface, the reactivity of adatoms towards O_2 has received much attention, because the latter ones are more reactive than restatoms.[5,6,7,8] The

nomenclature of oxidized adatoms was coined by Schubert *et al.*:[9] the *n×ins-paul* (respectively the *n×ins-ad)* corresponds to *n* oxygen atoms inserted into the backbonds of an adatom capped by an oxygen molecule (respectively a single oxygen atom). According to calculations,[10,11] the *3×ins-ad* "SiO_4" unit is metastable, the on-top oxygen (*ad*) moving in between the first and second plane and becomes tri-coordinated (*tri*), to give a *3×ins-tri* structure. Assuming that each adatom of the 7×7 surface cell is bonded to four oxygen atoms (*3×ins-ad* or *3×ins-tri*), the maximum oxygen coverage due to oxidized adatoms would be 12×4/49 ML (~1 ML). Given the present O coverage of 1.4 ML, oxidation involves other sites than the adatoms. This is in agreement with an O 1s XPS analysis of the Si(111)-7×7 surface exposed to O_2 at 110 K up to 3.6 L concludes to the reaction of restatom sites at high coverage.[8]

The O 1s spectrum fitting of figure S2 (b) can be interpreted according to references 6,7,11, although they concern specifically the regime of adatom oxidation. The main, lower binding energy component at 531.7 eV can be attributed to the "*ins*" species in the adatoms, and possibly to O inserted in the backbonds of the restatoms. The components at higher binding energy (at +0.8 eV and +1.6 eV from the main line), could arise from the "*tri*" and "*ad*" species, respectively of the adatoms. The main information gained from the O 1s spectrum is the complete absence of components at +3.1 and -4.4 eV from the main peak attributed to metastable molecular species (the *paul* oxygen of the *ins-paul* species).[6]

S3. O 1s monitoring during thermal de-oxidation

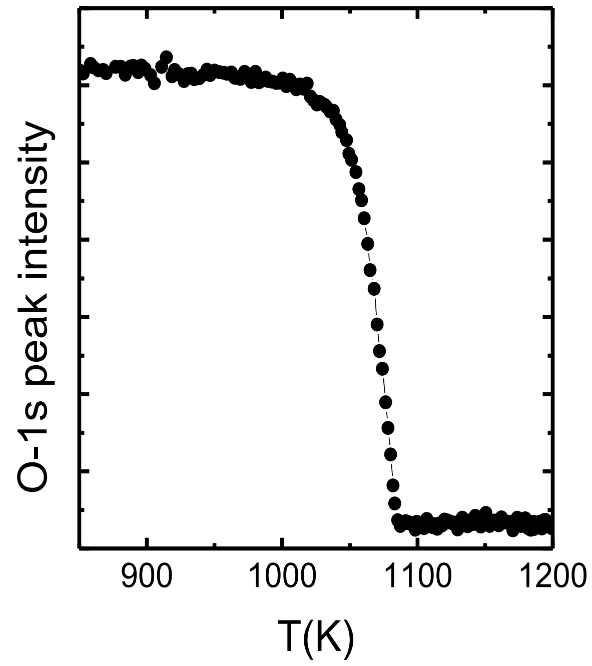

Figure S3. *O 1s intensity (hv=640 eV) plotted as a function of temperature for the slowest heating schedule (v_{av} =2.5 K.s^{-1}). The desorption temperature interval using Si^{4+} (Si 2p) as a proxy is 1049-1084 K.*

S4. Si 2p fitting procedure

An Igor procedure was written to fit sequentially all the spectra measured as a function of time. The Si 2p core-level is actually a doublet with a spin-orbit splitting of 0.61 eV and a $2p_{3/2}$:$2p_{1/2}$ branching ratio of 2. Therefore, the measured Si 2p spectra are fitted with six doublets corresponding to the main bulk component Si^0 the four silicon oxidation states Si^{n+}, the Si restatom (RA) component of the 7×7 DAS reconstructed surface. Si^0 encompasses the various components the various components of the clean surface except the RA component, and the bulk and interfacial Si^0 components in the case of the thermal Si/SiO_2 interface.[12] The doublets were built with a Voigt functions, with a fixed Lorentzian width of 50 meV. The fit parameters are given in Table S1. The bulk component BE energy varies when the sample is heated and deoxidized due to changes in the position of the Fermi level with respect to the valence band maximum. This position is let free and increases typically by ~0.2 eV. The RA and the oxide state components are shifted in energy with respect to the main bulk component Si^0 by the

values indicated in Table S1. In the sequential fitting process the parameters optimized for spectrum number i were used as the initial guess for spectrum $i + 1$.

	BE Shift from main Si^0 peak (eV)	Gaussian Width (eV)
Elemental silicon components	0	$0.50 - 0.40$
Si^{1+}	0.95 ± 0.01	$0.70 - 0.60$
Si^{2+}	1.79 ± 0.04	$0.80 - 0.70$
Si^{3+}	2.65 ± 0.15	$0.95 - 0.80$
Si^{4+}	3.5 ± 0.10	$1.17 - 1.10$
RA (surface)	-0.8 ± 0.1	$0.50 - 0.40$

Table S1: *Parameters (BE and Gaussian widths) used in the fitting procedure. The BE position of the RA component and of the oxide states are relative to that of the bulk peak. The Lorentzian width is 50 meV. The overall experimental resolution was 125 meV.*

S5. Avrami kinetics

Within the Avrami model, the fraction θ of untransformed matter is expressed as:

$$\theta = \exp(-\alpha_{ex}) \qquad (1)$$

where α_{ex} is the extended transformed phase fraction, that is, the fractional surface the transformed phase would acquire if the overlap among the growing nuclei were disregarded.[13] One assumes that the system is two-dimensional and that the reaction is limited at the periphery of the growing transformed phase (interface controled growth) that forms circular patches before overlapping. Then θ has the general form:

$$\theta = \exp(-\beta^n) \qquad (2)$$

where β is the transformation path and n the Avrami exponent.

The Avrami exponent n is equal to 2 in the case of prexisting or immediate nucleation (the nucleation rate is a Dirac's delta, also called site saturation) *and n* is equal to 3 in the case of a constant nucleation rate.

For isothermal transformations:

$\beta = kt$ (3)

where k is a rate constant (1/time) and t the time

k follows an Arrhenian temperature dependence:

$k = k_0 \exp(-E_{eff}/k_B T)$ (4)

where k_0 is the preexponential factor and E_{eff} the effective activation energy.

For $n = 2$, $k_0$writes as:

$k_0^2 = N\pi v_0^2$ (5a)

where N is the surface density (1/area) of nuclei and v_0 is the void radius growth velocity prefactor (length/time). In that case $E_{eff} = E_G$ i.e. the activation energy of the clean void growth.

For $n = 3$, $k_0$writes as:

$k_0^3 = (\pi/3) \times \dot{N} \times v_0^2$ (5b)

where $\dot{N}$ is the nucleation rate (1/(area × time)) and v_0 the growth velocity. In this case $E_{eff} = (E_N + 2E_G)/3$, where E_N is the nucleation activation energy.

Intermediate cases exist for which the surface density is neither a constant (zero nucleation rate) nor a linearly (infinitely) increasing function of time (constant nucleation rate). For a nucleation rate decreasing with increasing nuclei formation (N=-cst×n(t) where n(t) is the nuclei density) the Avrami formalism can be applied leading to intermediate values of *n*, see Kempen et al..[13]

For non-isothermal transformations, the Avrami formalism stands[14] provided that the transformation path $\beta = k \times t$ is replaced by:

$\beta(t) = \int_0^t k_0 \exp(-\frac{E_{eff}}{k_B T(t')})dt'$ (6)

where $T(t')$ is a function of time t'.

In the present case the temperature is not a linear function of time. Good fits of the temperature ramps can be obtained using the following formula:

$T(t) = T_m(1 - \exp(-\frac{\varphi}{T_m}t))$ (7)

where φ is a temperature rate (*K/s*) and T_m a saturation temperature.

A typical fit of $T(t)$ is shown in Figure S4.

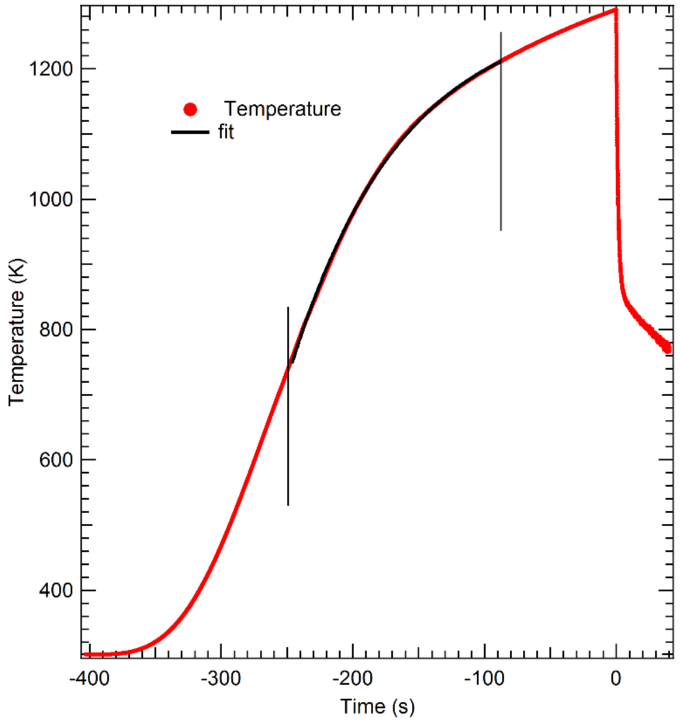

Figure S4. Temperature (K) versus time (s) curve (red) for the heating schedule #1 (τ_{ramp}=400 s) enabling the determination of parameters T_m (1295.4 K) and φ (15.3 K.s^{-1})) via a fit (black curve) taken in the time interval indicated by the two vertical bars. The pyrometer functions from a temperature of 600 K. Lower temperatures are only indicative (extrapolation from the T(t) curve above 600 K).

From (7) one can calculate the temperature rate that takes a simple analytical form

$\frac{dT(t)}{dt} = \varphi \times \frac{(T_m - T(t))}{T_m}$ with $T_m > T$ (8)

Using (6) and (8), the transformation path $\beta(t)$ can be expressed as a function of temperature T as:

$\beta(T) = \frac{k_0 T_m}{\varphi} \int_{T_{ini}}^{T} \frac{\exp(-\frac{E_{eff}}{k_B T'})}{(T_m - T')} dT'$ where T_{ini} is the initial temperature.

As T_{ini} is the room temperature, a temperature at which the de-oxidation process is blocked and thus $\beta(T < T_{ini}) \approx 0$. Therefore to an excellent approximation $\beta(T)$ can be rewritten as:

$$\beta(T) \approx \frac{k_0 T_m}{\varphi} \int_0^T \frac{\exp(-\frac{E_{eff}}{k_B T'})}{(T_m - T')} dT'$$

By integration one finds that:

$$\beta(T) = \frac{k_0 T_m}{\varphi}\left\{-\exp\left(-\frac{E_{eff}}{k_B T_m}\right)\mathrm{Ei}\left(-\frac{E_{eff}}{k_B}\left(\frac{1}{T}-\frac{1}{T_m}\right)\right)+\mathrm{Ei}\left(-\frac{E_{eff}}{k_B T}\right)\right\} \quad (9)$$

where Ei is the exponential integral $\mathrm{Ei}(x) = -\int_{-x}^{\infty}\frac{e^{-t}}{t}dt$

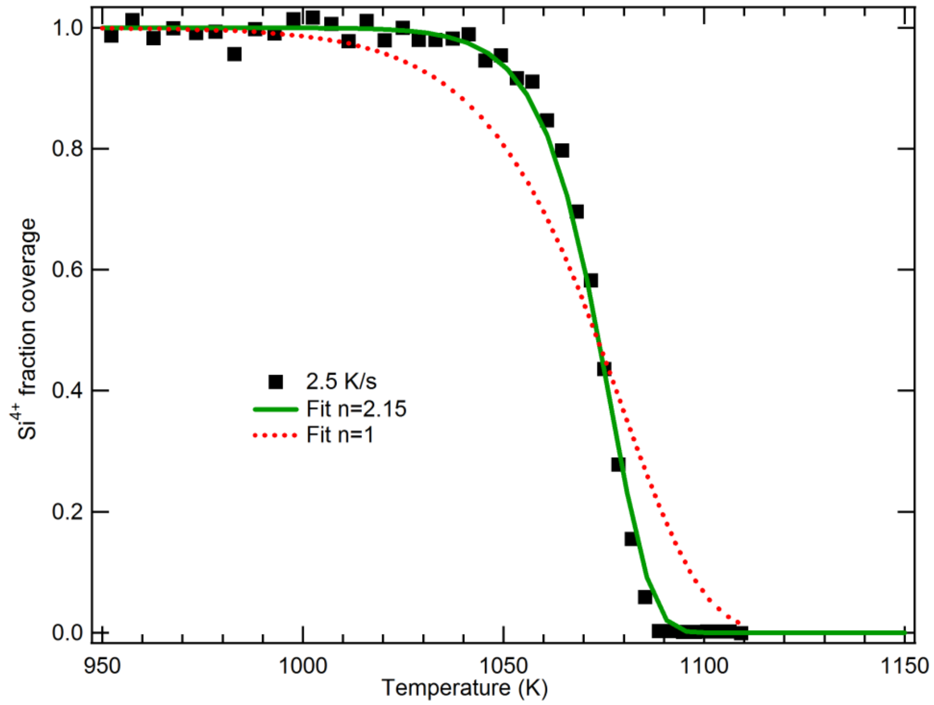

Figure S5. *Fits of the Si^{4+} desorption curve (temperature schedule (2.5 $K.s^{-1}$)) with n fixed to 1 (E_{eff}=4.48 eV, k_0=10^{20} s^{-1}) and n equal to 2.15 (E_{eff}=4.12 eV, k_0=2.4×10^{18} s^{-1}).*

The sensitivity of the fitting method was tested by imposing the value of the Avrami coefficient, letting the other two parameters (E_{eff} and k_0) free. The Si^{4+} desorption curve using the slower heating schedule (2.5 $K.s^{-1}$) is taken as an example, see figure S5. The green continuous curve corresponds to the case for which n is fixed to 2.15 (n exactly equal to 2 corresponds to an instantaneous nucleation in a two-dimensional system). The fitting is very satisfactory. On the other hand, the red dashed curve is the best fit obtained with n=1. The fit is clearly unsatifactory. n equal to 1 correspond to a first order reaction, as from equation (2)and (3) (isothermal case), $\ln(\theta) = -kt$ and $\frac{d\theta}{dt} = -k\theta$. It is clear that a first order reaction model, appropriate for homogeneous kinetics, cannot describe correctly the present desorption curves, although it is common practice to force data from a heterogeneous system into order-based models (see Ref. [15]). The choice of n (and hence of the desorption process model)

has a sizeable influence on the value of the (E_{eff}, k_0) couple, that is equal to (4.48 eV, 10^{20} s^{-1}) for n=1 and (4.12 eV, 2.4×10^{18} s^{-1}) for n=2.15 (note the compensation effect).

References:

1. Bergeard, N. *et al.* Time-resolved photoelectron spectroscopy using synchrotron radiation time structure. *J. Synchrotron Radiat.* **18,** 245–250 (2011).
2. Nambu, A. *et al.* An ultrahigh-speed one-dimensional detector for use in synchrotron radiation spectroscopy: first photoemission results. *J. Electron Spectros. Relat. Phenomena* **137–140,** 691–697 (2004).
3. Mathieu, C. *et al.* Molecular Staples on Si(001)-2 × 1: Dual-Head Primary Amines. *J. Phys. Chem. C* **113,** 11336–11345 (2009).
4. Nishimura, T., Hoshino, Y., Namba, H. & Kido, Y. Initial oxidation of Si(111)-7×7 surfaces studied by photoelectron spectroscopy combined with medium energy ion scattering. *Surf. Sci.* **461,** 146–154 (2000).
5. Comtet, G., Hellner, L., Dujardin, G. & Ramage, M. J. Adsorption of O2 on Si(111)7 × 7 compared at 300 and 30 K. *Surf. Sci.* **352–354,** 315–321 (1996).
6. Sakamoto, K., Zhang, H. M. & Uhrberg, R. I. G. Observation of two metastable oxygen species adsorbed on a Si(7×7) surface: Reinterpretation of the initial oxidation process. *Phys. Rev. B* **68,** 75302 (2003).
7. Sakamoto, K., Zhang, H. M. & Uhrberg, R. I. G. Photoemission study of metastable oxygen adsorbed on a Si(111)−7×7 surface. *Phys. Rev. B* **70,** 35301 (2004).
8. Wang, S., He, J., Zhang, Y. & Xu, G. Q. Adsorption of O2 and CO2 on the Si(111)-7×7 surfaces. *Surf. Sci.* **606,** 1387–1392 (2012).
9. Schubert, B., Avouris, P. & Hoffmann, R. A theoretical study of the initial stages of Si(111)–7×7 oxidation. I. The molecular precursor. *J. Chem. Phys.* **98,** 7593–7605 (1993).
10. Lee, S.-H. & Kang, M.-H. Electronic and vibrational properties of initial-stage oxidation products on Si(111)-7×7. *Phys. Rev. B* **61,** 8250–8255 (2000).
11. Lee, S.-H. & Kang, M.-H. Origin of O 1s Core-Level Shifts on Oxygen Adsorbed Si(111)−7×7. *Phys. Rev. Lett.* **84,** 1724–1727 (2000).
12. Jolly, F., Rochet, F., Dufour, G., Grupp, C. & Taleb-Ibrahimi, A. Oxidized silicon surfaces studied by high resolution Si 2p core-level photoelectron spectroscopy using synchrotron radiation. *J. Non. Cryst. Solids* **280,** 150–155 (2001).
13. Kempen, A. T. W., Sommer, F. & Mittemeijer, E. J. Determination and interpretation of isothermal and non-isothermal transformation kinetics; The effective activation energies in terms of nucleation and growth. *J. Mater. Sci.* **37,** 1321–1332 (2002).
14. Farjas, J. & Roura, P. Modification of the Kolmogorov–Johnson–Mehl–Avrami rate equation for non-isothermal experiments and its analytical solution. *Acta Mater.* **54,** 5573–5579 (2006).
15. Engstrom, J. R., Bonser, D. J., Nelson, M. M. & Engel, T. The reaction of atomic oxygen with Si(100) and Si(111). *Surf. Sci.* **256,** 317–343 (1991).
